# Augmented ustekinumab dosing is needed to achieve clinical response in patients with anti-TNF refractory pediatric Crohn’s disease: a retrospective chart review

**DOI:** 10.12688/f1000research.22673.2

**Published:** 2021-08-09

**Authors:** Phinga Do, John Andersen, Ashish Patel, Gaith Semrin, Luis Sifuentes-Dominguez, Phuong Luu, Bhaskar Gurram

**Affiliations:** 1 Department of Pediatrics, Children’s Health Medical Center Dallas, Dallas, TX, 75235, USA; 2 Department of Pediatric Gastroenterology, UT Southwestern Medical Center, Dallas, TX, 75235, USA

**Keywords:** Ustekinumab, Pediatric Crohn's disease, anti-TNF-refractory Crohn's disease, Inflammatory bowel disease, Therapeutic drug monitoring, Clinical response

## Abstract

**Background: **Ustekinumab is a monoclonal antibody that inhibits interleukins 12 and 23. It is approved for treatment of Crohn’s disease (CD) in adults; however, there is a paucity of data regarding its use in pediatric CD. We describe our experience using ustekinumab in anti-TNF refractory CD pediatric patients.

**Methods: **We performed a retrospective chart review on pediatric patients with CD who were started on ustekinumab from January 2016 to November 2018. We collected patient’s clinical history, previous treatment history, surgeries related to CD, disease severity, as measured by abbrPCDAI, and endoscopic severity as recorded by SES-CD before and after ustekinumab.

**Results: **We identified 10 patients with CD who were started on ustekinumab due to non-response to currently approved agents. Seven patients needed augmented maintenance dosing every 4-6 weeks to achieve clinical response or remission. Six of these seven patients had therapeutic drug monitoring during the course of treatment, with five patients showing subtherapeutic drug levels of <4.5 μg/mL while on standard maintenance dosing every 8 weeks, and four patients showing therapeutic drug levels of >4.5 μg/mL on augmented dosing interval. The remaining three patients were on standard maintenance dosing for the duration of treatment.

**Conclusion:** In this retrospective chart review, 7 out of 10 patients with anti-TNF refractory pediatric-onset CD required augmented maintenance doses of ustekinumab to achieve clinical response or remission. A prospective study is needed to define appropriate ustekinumab dosing and interval in management of pediatric CD.

## Introduction

Together, ulcerative colitis and Crohn’s disease (CD) make up inflammatory bowel disease (IBD), an autoimmune-mediated process of unclear etiology. The global incidence of pediatric IBD has been rising rapidly, with the highest incidence of CD being in Europe at 23/100000 person years and North America at 15.2/100000 person years ^1– 3^. Earlier onset of IBD is associated with higher impact on growth and development, more aggressive disease course, and increased need for immunomodulators ^4^. Anti-tumor necrosis factors (anti-TNFs) form the forefront of management of patients with CD who do not respond to steroids and immunomodulatory medications ^5^.

Among pediatric patients with CD who are started on anti-TNF treatments, about 10–25% do not respond to it (primary non-responders) ^6^. Of those who initially respond, loss of response and adverse effects limit duration of therapy. At 1, 3, and 5 years after therapy initiation, the probability of patients remaining on infliximab is only 0.87, 0.74, and 0.67, respectively (secondary non-responders) ^7^. Thus, there is a significant need for novel therapies for management of CD.

Among the newer biologics approved for treatment of CD is ustekinumab, a human immunoglobulin G1 kappa monoclonal antibody that binds with high affinity to the p40 subunit of human interleukin (IL)-12 and IL23. Ustekinumab prevents IL12 and IL23 bioactivity by preventing their interaction with their cell surface receptor protein IL12Rb1. Through this mechanism of action, ustekinumab effectively neutralizes IL12 (Th1)- and IL23 (Th17)-mediated cellular responses. It has recently been approved for the treatment of moderate to severe active CD in adults ^8^. However, data on usage of ustekinumab in management of pediatric Crohn’s disease is limited to small case series ^9– 11^. Here we describe our experience on using ustekinumab for management of TNF-refractory pediatric CD.

## Methods

We performed a retrospective chart review on 10 pediatric CD patients who failed anti-TNF therapy and were treated with ustekinumab between January 2016 and November 2018. Ustekinumab levels and antibodies were done either by Miraca or Inform Diagnostics. Both these labs used automated enzyme linked immunosorbent assay (ELISA) assay for simultaneous antibody and drug levels.

This study was approved by the Institutional Review Board (IRB) of Dallas Children’s Hospital (study #25338). Request for waiver of patient/guardian consent for this study was approved by the IRB.

### Data collection

We collected baseline demographic data, disease phenotype based on Paris Classification ^12^, disease related complications, previous treatment history, and reason for changing therapy.

To assess clinical response to ustekinumab, we calculated the Abbreviated Pediatric Crohn’s Disease Activity Index (abbrPCDAI) prior to starting therapy, 2–3 months after therapy initiation, and at the last office visit before conclusion of the study ^13^. When no office visits were available immediately prior to treatment initiation, telephone and email encounters were used to assess patients’ clinical symptoms to calculate abbrPCDAI. Where possible we also calculated the Simple Endoscopic Score for Crohn’s Disease (SES-CD) before and after treatment initiation ^14^. Body Mass Index (BMI) before and after treatment was collected.

Laboratory measurements, which include hematocrit, C-reactive protein (CRP), and albumin, were also collected before and after treatment initiation. We also looked at the trough ustekinumab levels where available in relation to dose and response to therapy.

### Data analysis

Patients are categorized as anti-TNF primary non-responders if there’s no clinical response during therapy induction, and secondary non-responders if there’s loss of response during maintenance phase ^15^. Based on abbrPCDAI, clinical response is defined as ≥15 points reduction, and clinical remission is defined as <10. We define sustained clinical remission as abbrPCDAI of <10 with no subsequent elevation in AbbrPCDAI as of the last visit. Disease severity is categorized as follows: severe >25; moderate 16–25; mild <16. We use the following SES-CD cutoff to define disease severity: remission 0–2; mild 3–6; moderate 7–15; and severe >16 ^16^. Endoscopic response is defined as ≥50% decrease in SES-CD score compared to baseline ^17^. Based on previous studies we used a target ustekinumab trough level of >4.5 µg/mL ^18^.

## Results

Patients’ age at initial diagnosis ranged from 2 to 14 years (median age of 9.5 years). Age at initiation of ustekinumab ranged from 9 to 19 years (median age of 14.5 years). Duration of disease ranged from 3 to 14 years (median duration of 6.5 years). Table 1 summarizes patients’ demographic, disease phenotype at diagnosis, extraintestinal manifestations, disease related surgeries, treatment history, and reasons for changing therapy to ustekinumab. Of note, patient one was diagnosed at two years of age, putting him in the very early onset IBD (VEO-IBD) category. Even though VEO-IBD is a phenotypically distinct group, there’s still significant overlap in behavior and response to therapy between VEO-IBD and conventional-onset IBD ^19^. All 10 patients in our cohort were refractory to anti-TNF therapy.

**Table 1.** Baseline characteristics of patients included in the study.

Patient	Gender	Age at diagnosis	Current age	Age at ustekinumab initiation	Paris Classification at diagnosis	abbrPCDAI at diagnosis	Extra-intestinal manifestations	Perianal disease	Surgery	Past immunomodulators	Past anti-TNF therapy	Other past biologics	Reason for switching to ustekinumab
1	M	2	11	9	A1aL3L4aB1pG1	30	None	Yes	Ileocececto- my with ileostomy	Thiopurines	Infliximab, adalimumab	NA	Secondary loss of response to infliximab and adalimumab. No level or antibody obtained
2	M	13	16	15	A1bL3L4aB1G1	40	None	No	No	Methotrexate	Infliximab, adalimumab	Vedolizumab	Secondary anti-TNF non-responder, antibody development leading to transfusion reaction
3	F	10	18	17	NA	NA	Arthritis	No	Complete colectomy	Thiopurines, methotrexate	Infliximab, adalimumab	NA	Primary anti-TNF non-responder. No antibody detected
4	F	9	16	14	A1aL3L4aB1G1	30	None	No	Distal loop ileostomy	Thiopurines, methotrexate	Infliximab, adalimumab	Vedolizumab	Secondary anti-TNF non- responder. Developed antibody to inflximab. Poor response despite good level and no antibody to adalimumab
5	F	9	13	11	A1aL3aB1pG1	35	None	Yes	No	Methotrexate	Infliximab, adalimumab	Vedolizumab	Primary lack of response to infliximab and adalimumab. No inflximab level; therapuetic adalimumab level with no antibody
6	M	11	17	14	A1bL1L4aB1G1	25	Arthritis	Yes	No	Thiopurines, methotrexate	Infliximab, adalimumab, certolizumab	NA	Secondary loss of response and psoriasis with infliximab and adalimumab with antibody development
7	M	9	15	12	A1aL3L4aB1pG1	30	Fever, arthritis, episcleritis	Yes	No	NA	Infliximab, adalimumab	NA	Clinical secondary loss of response
8	M	14	19	17	A1bL3L4aB2G1	20	None	No	No	Thiopurines	Adalimumab	NA	Secondary loss of respone. No antibody, therapeutic level
9	F	7	21	19	A1aL3B1P	35	Arthritis, fever	Yes	No	Thiopurines, methotrexate	Infliximab, adalimumab	NA	Primary non response to alalimumab without drug level. Lymphoma on infliximab
10	F	10	18	16	A1bL3L4aB2G1	45	Arthritis, fever	Yes	No	Thiopurines, methotrexate	Infliximab, adalimumab	Vedolizumab	Psoriasis reaction with infliximab

EIM- extra intestinal manifestations. **AbbrPCDAI - abbreviated pediatric Crohn Disease activity index**.

Table 2 summarizes the ustekinumab induction and maintenance dose used in these patients. For induction, the dosing varied among patients with 7 out of 10 receiving induction doses per current recommendations ^20^ The remaining 3 patients received doses as described in Table 2

**Table 2.** Ustekinumab dosing, interval, levels, duration of therapy, and complications.

Patient	Induction (mg)	Maintenance (mg)	Initial interval (weeks)	Trough level (ug/ml)	Final maintenance dose (mg)	Final intervals (weeks)	Last trough level (µg/mL)	Therapy duration (weeks)	Complications on Ustekinumab
1	45x2	45	8	NA	90	8	NA	142	NA
2	520	90	8	undetectable	90	4	NA	59	NA
3	260	90	8	3.6	90	4	8	63	infusion reaction
4	240	90	6	0.8	90	4	7.1	71	NA
5	180	45x1, then 90	6	0.1	90	6	>10	73	NA
6 *	390	90	4	10	90	8	NA	16	skin abscess
7	90x3 Q4	90	8	1.1	90	6	NA	143	skin abscess
8	260	90	8	NA	90	8	NA	55	*Clostridium * *difficile* infection
9	390	90	8	NA	90	8	NA	36	NA
10	390	90	8	NA	90	8	NA	25	NA

*This patient was exposed to ustekinumab 2 years prior for a duration of 4 weeks

### Patients on augmented dose

**Figure 1.**
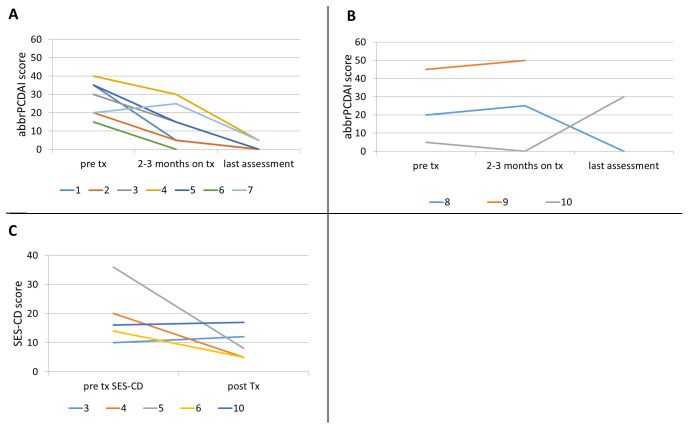
Clinical and endoscopic response. Abbreviated-pediatric Crohn’s disease activity index in patients with ( **A**) augmented ustekinumab dosing and ( **B**) Q8 dosing; ( **C**) simple endoscopic score-Crohn’s disease (SES-CD) pre and post-ustekinumab initiation.

Of the seven patients who received augmented maintenance doses, all seven showed clinical response, as shown in Figure 1 (patients 1–7), and all but one patient achieved sustained clinical remission as assessed by abbrPCDAI. One patient (patient 7) achieved remission after 5 months of specific carbohydrate diet (SCD) and ustekinumab at Q8 maintenance. He remained in remission for the next 17 months, then developed fatigue and bloody stool when diet was liberalized. His symptoms did not respond to reintroduction of SCD. However, he went into clinical remission when ustekinumab maintenance interval was changed to Q6 weeks.

Only 4 out of 10 patients had endoscopy before and after ustekinumab treatment. Of these, three patients showed endoscopic response and one showed worsening of SES-CD score ( Figure 1C). Of note, all 3 patients who showed endoscopic response received augmented dose. While no patient showed mucosal remission, mucosal inflammation did improve from severe to mild, severe to moderate, and moderate to mild in three patients.

**Figure 2.**
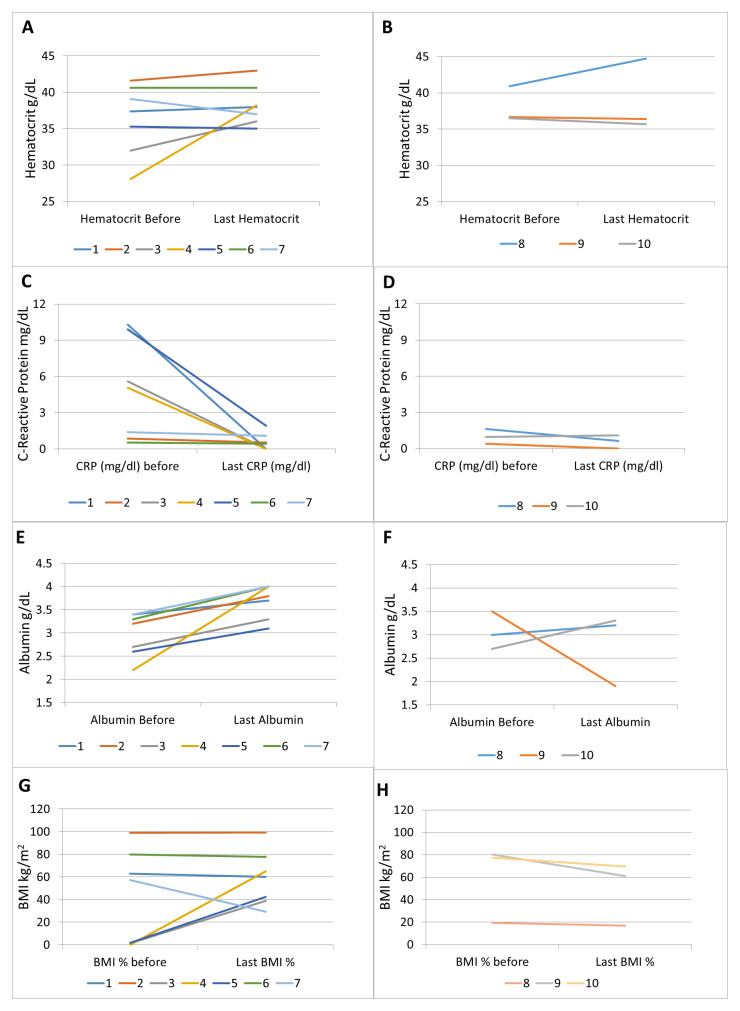
Laboratory and BMI response. Hematocrit in patients with ( **A**) augmented dosing and ( **B**) Q8 dosing; CRP in patients with ( **C**) augmented dosing and ( **D**) Q8 dosing; albumin in patients with ( **E**) augmented dosing and ( **F**) Q8 dosing; BMI in patients with ( **G**) augmented dosing and ( **H**) Q8 dosing.

Laboratory indices also improved in 6 out of 7 patients ( Table 3). The most significant and consistent improvements were seen in CRP and albumin ( Figure 2C–F). BMI improved significantly in patients with pre-treatment BMI below the 2 ^nd^ percentile. In patients with normal BMI (5 ^th^ to 85 ^th^ percentile), the BMI either decreased or showed small numerical improvement ( Figure 2G,H).

**Table 3.** laboratory and clinical response to ustekinumab.

Patient	Hematocrit Before	Last Hematocrit	CRP (mg/dl) before	Last CRP (mg/dl)	Albumin Before	Last Albumin	BMI % before	Last BMI %	abbrPCDAI pre tx	abbrPCDAI 2-3 months on tx	abbrPCDAI last assessment	pre tx SES-CD	post Tx SES-CD
1	37.4	38	10.3	<0.29	3.4	3.7	63	60	35	5	0	NA	NA
2	41.6	43	0.869	0.516	3.2	3.8	99	99.3	20	5	0	NA	NA
3	32	36	5.61	<0.29	2.7	3.3	1.31	38.9	30	15	NA	10	12
4	28.1	38.2	5.06	<0.29	2.2	4	0.15	65	40	30	5	20	5
5	35.3	35	9.91	1.92	2.6	3.1	1.88	42.3	35	15	0	36	8
6 *	40.6	40.6	0.52	0.43	3.3	4	79.8	77.8	15	0	NA	14	5
7	39.1	37	1.39	1.1	3.4	4	57.4	29.35	20	25	5	NA	NA
8	40.9	44.7	1.62	0.625	3	3.2	19.4	17	20	25	0	NA	NA
9	36.7	36.4	0.4	<0.29	3.5	1.9	80.4	61	45	50	NA	NA	NA
10	36.5	35.7	0.95	1.11	2.7	3.3	77.3	69.7	5	0	30	16	17

*Data collected for patient's second time on ustekinumab

Among the seven patients, five had ustekinumab trough level showing low or undetectable drug level when receiving medication at a 6 or 8 week intervals ( Table 2). Subsequently, four patients had escalation in frequency to Q4 and either achieved remission or clinical improvement. Following this change in interval, repeat drug levels for three patients were all therapeutic at 8, 7.1, and >10 μg/mL. Subsequently one of these three patients’ maintenance interval was decreased to Q6, and by the time of this study’s conclusion, a repeat level has not been obtained. Frequency was increased to Q6 in another patient, resulting in clinical remission. One of the patients was empirically started on a maintenance dose of Q4 interval and had trough levels of >10 μg/mL at 4 weeks. Frequency was subsequently changed to Q8, but a repeat drug level was not obtained ( Table 2).

### Patients on standard dose interval

Three of the 10 patients were on standard ustekinumab dosing. One patient had symptomatic duodenal stricture and obstruction, resulting in abdominal pain, vomiting, and weight loss. He underwent endoscopic stricture dilation 2 months prior to initiation of ustekinumab. After ustekinumab was started, he required two subsequent dilations in a 2-month period, but had subsequently been in remission for the next 5 months on high dose steroid. However, this was confounded by his family continuing 60mg prednisone daily for at least 4 months (2 months longer than prescribed). Unfortunately, there was no subsequent follow ups as he had transitioned to adult care. His hematocrit, CRP, and albumin all improved compared to levels prior to ustekinumab, while his BMI decreased slightly. The remaining two patients had disease worsening on ustekinumab, shown by serology and worsening abbrPCDAI. None of these three patients had their levels checked.

Complications observed while on ustekinumab included infusion reactions, such as low-grade fever, joint pain and vomiting within one week of infusion, and infections such as *Clostridium difficile*, influenza, and pneumonia. Of note, one patient developed perianal abscess within a few weeks of the first ustekinumab induction, requiring hospitalization and resulting in stopping therapy. Upon the second induction more than 1.5 years later, he again developed forearm abscess requiring hospitalization. However, his CD went into remission with ustekinumab. Work-up for immune deficiency was negative. He was later diagnosed with maturity-onset diabetes of the young.

## Discussion

Here we report 10 pediatric patients with CD refractory to currently approved medications including anti-TNFs, immunomodulators and some to vedolizumab, with seven showing a clinical response to ustekinumab treatment. The majority of our patients showed positive response to ustekinumab within the first 2–3 months of therapy and clinical remission by the time this study was concluded. Four of these seven patients had endoscopic data pre- and post-ustekinumab, out of which three showed an improvement as measured by SES-CD. In general, SES-CD score showed higher level of disease activity than abbrPCDAI, which is likely due to poor correlation between these two indices ^21^. Moreover, abbrPCDAI and SES-CD information were collected at different times in the treatment course, resulting in small differences in disease activities. CRP, albumin, and BMI showed the largest improvement, and hematocrit improved in all but two patients who responded to treatment.

To achieve clinical response and/or remission, 7 out of 10 patients needed augmented maintenance doses. Of note, one patient’s (patient 7) level of 1.1 μg/mL coincided with disease exacerbation and increase in maintenance frequency to Q6 resulted in clinical remission. However, more data from subsequent follow ups is needed to determine if disease activity corresponds to dosing frequency and trough level since he had no trough level during previous remission on SCD diet.

Among the three patients on standard dosing for the entire duration of treatment, only one achieved remission. He had ileocolonic as well as symptomatic gastroduodenal CD, which is a relatively rare manifestation and only affects about 2% of CD patients ^22^. There are currently no well- established treatment protocols for gastroduodenal CD, and despite treatments with corticosteroid, 6-MP, ASA, and anti-TNF agents, 31% of patients eventually require surgery ^23^. We cannot conclude if patient 8’s clinical improvement was secondary to ustekinumab or corticosteroids.

In a previous study by Battat *et al.* over 75% of adult CD patients needed Q4 week dosing for maintenance of clinical response ^18^. This study also demonstrated a positive association of biomarkers and endoscopic improvement with ustekinumab trough levels >4.5 μg/mL ^18^. In addition, in a case series of three adult CD patients, Park et al demonstrated an ability to recapture response by dose escalation among patients who lost response to standard ustekinumab dosing regimen ^24^. Several observational studies in pediatric IBD patients demonstrate ustekinumab’s efficacy in inducing and maintaining remission ^20, 25^. While the majority of patients in both studies had dose frequency escalation, the decision was made based on patient’s lack of clinical response. Even though ustekinumab concentration and antibody level was measured for more than half of the 52 patients studied by Dayan *et al*., the author noted that there was no timing consistency for these measurements, and there was no correlation between dosing escalation and clinical remission. In contrast, among the 7 patients who required dose escalation in our study, 6 had subtherapeutic drug level on Q6 and Q8 dosing interval, which corresponded with poorly controlled disease activities. All these patients showed clinical response to changing the dosing interval. On the two patients who were on standard dosing interval (Q8 weeks), therapeutic drug monitoring (TDM) was not performed and thus we cannot determine if treatment failure was due to subtherapeutic dosing or a primary non-response to ustekinumab. This study demonstrates that TDM can be used to guide dose frequency escalation in patients who show no clinical response or lack of clinical response to ustekinumab. A larger, randomized trial is needed to confirm the role of ustekinumab TDM in pediatric CD patients. Thus, based on our experience and existing literature, we recommend proactively checking trough levels 4 weeks after maintenance therapy initiation to guide dosing frequency early in the treatment course or to consider reactively checking levels and augmenting maintenance dosing interval in patients with sub-optimal or poor response to standard dosing.

Serious adverse effects were rare among our patients despite shorter dosing intervals. Only two patients developed recurrent infections and required hospitalization while on ustekinumab. Even though the patient who was hospitalized for abscesses also had other comorbidities such as maturity onset diabetes of the young, acne, skin picking, psoriasis, and was previously hospitalized twice for recurrent abscesses on certolizumab, ustekinumab could not be ruled out as a cause of these infections. We did not observe any serious infections or cancers in the remaining eight patients, suggesting that ustekinumab is relatively well tolerated even at a higher frequency in pediatric patients.

### Limitations

Our study is limited by its small size and retrospective nature. Furthermore, induction and maintenance doses were not uniform among all patients. TDM was only performed on six patients, and follow-up trough for five out of those six patients have not been obtained after changes in dosing frequency.

Only six patients had pre- and post-treatment endoscopy, and two of those patients had intestinal surgeries, which might have altered SES-CD scores. Even though abbrPCDAI and other laboratory workups were helpful to correlate disease activities, fecal calprotectin should be added to further assess inflammation.

## Conclusion

In this retrospective chart review, 7 out of 10 patients with anti-TNF refractory pediatric-onset Crohn’s disease required augmented maintenance doses of ustekinumab to achieve clinical response or remission as measured by abbrPCDAI. The remaining three patients on standard maintenance doses either did not respond or had confounding factors affecting clinical response. Further large, randomized studies with closer therapeutic drug monitoring are needed to assess the relationship between dosing interval, trough levels, and clinical response in the pediatric population. Longer follow up is also needed to assess response once ustekinumab has reached therapeutic level.

## Data availability

### Underlying data

Figshare: Table 1. Baseline characteristics of patients included in the study, https://doi.org/10.6084/m9.figshare.14681013 ^26^.

Figshare: Table 2. Ustekinumab dosing, interval, levels, duration of therapy, and complication, https://doi.org/10.6084/m9.figshare.14681028 ^27^.

Figshare: Table 3 Clinical response of anti-TNF refractory pediatric Crohn Disease patients on ustekinumab.csv, https://doi.org/10.6084/m9.figshare.14681040 ^28^.

Data are available under the terms of the Creative Commons Zero “No rights reserved” data waiver (CC0 1.0 Public domain dedication).
