# Supplementary material for: Augmented ustekinumab dosing is needed to achieve clinical response in patients with anti-TNF refractory pediatric Crohn’s disease: a retrospective chart review
Source: F1000Res. 2021 Aug 9;9:316. Originally published 2020 Apr 30. [Version 2] doi: 10.12688/f1000research.22673.2 (PMC8408544; doi:10.12688/f1000research.22673.2)
Supplement: Peer review history

# Peer review: Reviewer response for version 2

**DOI:** 10.5256/f1000research.57245.r91519

**Published:** 2021-08-31

**Authors:** Richard Kellermayer

**Affiliations:** 1 Department of Pediatrics, Baylor College of Medicine, Houston, TX, USA; 2 Section of Pediatric Gastroenterology, Texas Children's Hospital, Houston, TX, USA; 3 USDA/ARS Children's Nutrition Research Center, Houston, TX, USA

The authors have addressed all my concerns appropriately.

Is the work clearly and accurately presented and does it cite the current literature?

Partly

If applicable, is the statistical analysis and its interpretation appropriate?

Partly

Are all the source data underlying the results available to ensure full reproducibility?

Partly

Is the study design appropriate and is the work technically sound?

Yes

Are the conclusions drawn adequately supported by the results?

Yes

Are sufficient details of methods and analysis provided to allow replication by others?

Partly

Reviewer Expertise:

Inflammatory bowel disease, Crohn's disease, ulcerative colitis, molecular genetics, epigenetics, nutrition, developmental origins of disease, recurrent Clostridioides difficile infection, fecal transplantation, microbiome, microbial therapeutics.

I confirm that I have read this submission and believe that I have an appropriate level of expertise to confirm that it is of an acceptable scientific standard.

# Peer review: Reviewer response for version 1

**DOI:** 10.5256/f1000research.25033.r62949

**Published:** 2021-04-12

**Authors:** Abdul Elkadri

**Affiliations:** 1 Medical College of Wisconsin, Milwaukee, WI, USA

This study reviews retrospectively the experience of one pediatric institution with ustekinumab in Crohn's disease patients. This is an understudied population with paucity of data. Interestingly, it showed good response in 7/10 patients, but also showed that there was a need for shortened intervals of dosing. This shortened interval of dosing was shown previously in one of the adult studies cited and suggests that there needs to be more studies to support this shortened interval. The review is limited by the lack of comprehensive endoscopic data for all patients, and by the lack of comprehensive data for therapeutic drug monitoring to try to prove the hypothesis that shortened intervals lead to remission and clinical response (with resulting increased drug levels). The retrospective nature of this paper makes the data limited in its ability to prove the hypothesis, but the data is still reviewed well and with details all available to show what the authors have stated. The population is somewhat heterogeneous in nature, limiting the ability to identify a subpopulation where the response is higher. 

 Due to the small number of patients, the manuscript is by default limited to a descriptive review which the authors have done properly in this manuscript. The data needs to be published to expand the available literature on this medication in this population. 

 The current version of the manuscript is appropriate for indexing.

Is the work clearly and accurately presented and does it cite the current literature?

Yes

If applicable, is the statistical analysis and its interpretation appropriate?

Not applicable

Are all the source data underlying the results available to ensure full reproducibility?

Yes

Is the study design appropriate and is the work technically sound?

Yes

Are the conclusions drawn adequately supported by the results?

Yes

Are sufficient details of methods and analysis provided to allow replication by others?

Yes

Reviewer Expertise:

Pediatric inflammatory bowel Disease

I confirm that I have read this submission and believe that I have an appropriate level of expertise to confirm that it is of an acceptable scientific standard.

# Peer review: Reviewer response for version 1

**DOI:** 10.5256/f1000research.25033.r62953

**Published:** 2020-05-18

**Authors:** Richard Kellermayer

**Affiliations:** 1 Department of Pediatrics, Baylor College of Medicine, Houston, TX, USA; 2 Section of Pediatric Gastroenterology, Texas Children's Hospital, Houston, TX, USA; 3 USDA/ARS Children's Nutrition Research Center, Houston, TX, USA

Phinga Do and colleagues, with the leadership of Bhaskar Gurram, give a nice summary on 10 pediatric Crohn’s disease patients, refractory to anti-TNF biologic therapy, who were treated with ustekinumab. Such real life observations on the off label use of new biologic agents in pediatric inflammatory bowel disease (IBD) patients are rather important. Their conclusions coincide with our subjective observation that pediatric IBD patients require a more intensified use of ustekinumab than the manufacturer’s guidance, in order to achieve drug levels that have been found to associate with improved outcomes in adult patients. There are a couple of recommendations, which may further improve the manuscript.

1. In the Methods section, please clarify which laboratory and what methodology was used (especially for ustekinumab therapeutic drug monitoring) for the laboratory measurements discussed in the paper.
2. Very early onset (VEO) IBD is more and more considered a disease category of its own within IBD. Consider excluding the VEO-IBD (patient 1) case from this cohort, or highlight the case as a variant.
3. Excluding the brief 4 week course of patient 6 from the table and discussion is recommended. The treatment course was too short to make any conclusions. The comment below the figure is important to keep, however, to clarify that patient 6 was exposed to ustekinumab 2 years prior to the treatment course examined.
4. In Table 1, or in a separate table, it would be useful to clarify the type of anti-TNF failure for each patient and each anti-TNF biologic, respectively. Primary non-response could be due to rapid antibody development or primary biological non-response. The same is true for secondary non-response (i.e. late development of antibodies, or immune pathway shift). It is fine to state unknown/clinical if level and antibody for the anti-TNF agent was not examined.
5. In the results, consider omitting the complicated description of dosing, since Table 2 describes that in detail.
6. Both figures are complicated, and the results those depict could be easily described and followed in writing within the Results section. Omitting the figures is recommended.
7. The clinical outcomes are difficult to follow based on the separation of the patients by augmented ustekinumab treatment versus not. Separating the patients by responders vs. non-responders, and examining ustekinumab dosing differences between the 2 groups is recommended. If I am not mistaken, and even if the patient 1 (VEO-IBD) case is excluded, there were 7 responders (6 on intensified ustekinumab therapy) and 2 non-responders (all on conventional ustekinumab treatment). These results, if compared by Chi squared testing, significantly favor (p=0.0233) treatment intensification in order to achieve clinical response.
8. There are parts of the discussion, which continue specific patient outcome descriptions. This should be moved to the Results section. The discussion should only draw general conclusions from what has been presented in the results and compare the findings to the existing literature.
9. Include in the discussion, more recent similar studies, such as the ones below, and highlight the novelties of the case series presented within this work compared to other pediatric cohorts:
   
  1. Real World Experience With Ustekinumab in Children and Young Adults at a Tertiary Care Pediatric Inflammatory Bowel Disease Center. Dayan JR *et al.* J Pediatr Gastroenterol Nutr. (2019) ^1^
  2. Ustekinumab for the treatment of refractory pediatric Crohn's disease: a single-center experience. Cohen A, Ahmed N, Sant'Anna A.. Intest Res. 2020 Apr 22. ^2^

Is the work clearly and accurately presented and does it cite the current literature?

Partly

If applicable, is the statistical analysis and its interpretation appropriate?

Partly

Are all the source data underlying the results available to ensure full reproducibility?

Partly

Is the study design appropriate and is the work technically sound?

Yes

Are the conclusions drawn adequately supported by the results?

Yes

Are sufficient details of methods and analysis provided to allow replication by others?

Partly

Reviewer Expertise:

Inflammatory bowel disease, Crohn's disease, ulcerative colitis, molecular genetics, epigenetics, nutrition, developmental origins of disease, recurrent Clostridioides difficile infection, fecal transplantation, microbiome, microbial therapeutics.

I confirm that I have read this submission and believe that I have an appropriate level of expertise to confirm that it is of an acceptable scientific standard, however I have significant reservations, as outlined above.

## References

[1] : Real World Experience With Ustekinumab in Children and Young Adults at a Tertiary Care Pediatric Inflammatory Bowel Disease Center. *Journal of Pediatric Gastroenterology and Nutrition*.2019;69(1) : 10.1097/MPG.000000000000236261-6710.1097/MPG.0000000000002362PMC740844831058718

[2] : Ustekinumab for the treatment of refractory pediatric Crohn’s disease: a single-center experience. *Intestinal Research*.2020; 10.5217/ir.2019.0916410.5217/ir.2019.09164PMC810037532312033

# Response

**Published:** 2021-05-26

**Authors:** phinga do

**Affiliations:** UT Southwestern, USA

1. In the Methods section, please clarify which laboratory and what methodology was used (especially for ustekinumab therapeutic drug monitoring) for the laboratory measurements discussed in the paper. 
  All of the tests were done either by Miraca or Inform Diagnostics. Both these labs use Automated ELISA assay for simultaneous antibody and drug levels 
2. Very early onset (VEO) IBD is more and more considered a disease category of its own within IBD. Consider excluding the VEO-IBD (patient 1) case from this cohort, or highlight the case as a variant. 
  Although some patients with VEOIBD might behave differently than conventional-onset IBD patients (onset>10 years of age), there is still significant overlap with their disease behavior and response to therapy. Moreover, although the patient 1 included in the current study had onset at 2 years of age, he was started on ustekinumab at 9 years age. Inflamm Bowel Dis 2021 Feb 16;27(3):295-302. doi: 10.1093/ibd/izaa080. 
3. Excluding the brief 4-week course of patient 6 from the table and discussion is recommended. The treatment course was too short to make any conclusions. The comment below the figure is important to keep, however, to clarify that patient 6 was exposed to ustekinumab 2 years prior to the treatment course examined. 
  We will remove this part. Agree that it does not add much aside from noting that there’s prior exposure.
4. In Table 1, or in a separate table, it would be useful to clarify the type of anti-TNF failure for each patient and each anti-TNF biologic, respectively. Primary non-response could be due to rapid antibody development or primary biological non-response. The same is true for secondary non-response (i.e. late development of antibodies, or immune pathway shift). It is fine to state unknown/clinical if level and antibody for the anti-TNF agent was not examined. 
  Table 1 has been modified to include more specific reasons for anti-TNF failure.
5. In the results, consider omitting the complicated description of dosing, since Table 2 describes that in detail. 
  The results section has been edited to omit dosing descriptions.
    
6. Both figures are complicated, and the results those depict could be easily described and followed in writing within the Results section. Omitting the figures is recommended. 
  We believe that the figures compliment the text and some readers prefer just to look at the tables and pictures. They provide visual aid and show a clear trend of improvement for patients on augmented dosing.
7. The clinical outcomes are difficult to follow based on the separation of the patients by augmented ustekinumab treatment versus not. Separating the patients by responders vs. non-responders and examining ustekinumab dosing differences between the 2 groups is recommended. If I am not mistaken, and even if the patient 1 (VEO-IBD) case is excluded, there were 7 responders (6 on intensified ustekinumab therapy) and 2 non-responders (all on conventional ustekinumab treatment). These results, if compared by Chi squared testing, significantly favor (p=0.0233) treatment intensification in order to achieve clinical response. 
  We did organize it by responder vs non responder initially but found that organizing by augmented vs standard dosing shows a clearer trend. Moreover, what we want to point out to the readers through this paper is that most pediatric patients in our cohort needed higher dose than the standard dosing. In addition, we cannot say if patient 8 (the one with duodenal stricture) is an ustekinumab responder or not and will require 3 distinct categories (responder, non-responder, and indeterminant). All 7 responders were on augmented dose
8. There are parts of the discussion, which continue specific patient outcome descriptions. This should be moved to the Results section. The discussion should only draw general conclusions from what has been presented in the results and compare the findings to the existing literature. 
  The text has been reorganized.
9. Include in the discussion, more recent similar studies, such as the ones below, and highlight the novelties of the case series presented within this work compared to other pediatric cohorts:
  Edited to include these 2 studies.
